# Sesamol protects against liver fibrosis induced in rats by modulating lysophosphatidic acid receptor expression and TGF-β/Smad3 signaling pathway

**DOI:** 10.1007/s00210-022-02259-7

**Published:** 2022-06-01

**Authors:** Nesma A. Abd Elrazik, Mohamed El-Mesery, Mamdouh M. El-Shishtawy

**Affiliations:** Department of Biochemistry, Faculty of Pharmacy, Mansoura University, P.O. Box, Mansoura, 35516 Egypt

**Keywords:** Ductular reaction, Liver fibrosis, LPAR, Sesamol, TGF-β1/Smad3

## Abstract

The present study aimed to investigate the hepatoprotective effect of sesamol (SML), a nutritional phenolic compound obtained from sesame seeds, in liver fibrosis induced by thioacetamide (TAA) in rats and to explore the underlying mechanisms. Thirty-two male Sprague–Dawley rats were equally divided into four groups: control, TAA, TAA + SML 50 mg/kg, and TAA + SML 100 mg/kg groups. Liver functions and hepatic contents of glutathione (GSH) and malondialdehyde (MDA) were measured colorimetrically. Gene expressions of lysophosphatidic acid receptor (LPAR)-1 and -3, connective tissue growth factor (CTGF), transforming growth factor (TGF)-β1, small mothers against decapentaplegic (Smad)-3 and -7, α-smooth muscle actin (α-SMA), and cytokeratin 19 (CK19) were analyzed by qRT-PCR. Moreover, phosphorylated Smad3 (pSmad3) was quantified by ELISA. Additionally, TGF-β1, α-SMA, CK19, and vascular endothelial growth factor (VEGF) protein concentrations were semi-quantitatively analyzed by immunostaining of liver sections. SML treatment markedly improved liver index and liver functions. Moreover, SML protected against liver fibrosis in a dose-dependent manner as indicated by down-regulation of LPAR1, LPAR3, CTGF, TGF-β1/Smad3, and α-SMA expressions and a decrease in pSmad3 level, as well as an up-regulation of Smad7 expression. In addition, SML suppressed ductular reaction hinted by the decrease in CK19 expression. These results reveal the anti-fibrotic effect of SML against liver fibrosis that might be attributed to down-regulation of LPAR1/3 expressions, inhibition of TGF-β1/Smad3 pathway, and ductular reaction.

**Supplementary Information:**

The online version contains supplementary material available at 10.1007/s00210-022-02259-7.

## Introduction

Liver fibrosis is a complex inflammatory process that may progress to cirrhosis and hepatocellular carcinoma (HCC). Hepatic fibrosis demonstrates the wound healing process in response to liver injury which is characterized by an elevation in extracellular matrix (ECM) deposition around the sinusoidal cell layer in the space of Disse. The imbalance between ECM synthesis and degradation leads to an increase in the fibrotic matrix (Zhang et al. 2016; Roehlen et al. 2020).

Thioacetamide (TAA), a thiosulfur compound, is a hepatotoxic agent that is used in research to induce liver fibrosis and HCC by recurrent administration in experimental animals (Helmy et al. 2018; Ebrahim et al. 2018; Helmy et al. 2019; Shaker et al. 2021). TAA is metabolized to TAA sulfoxide and TAA disulfoxide metabolites that covalently bind to cellular macromolecules leading to an increase in reactive oxygen species (ROS) generation and acute centrilobular liver necrosis (Eraky et al. 2018; Nevzorova et al. 2020).

Lysophosphatidic acid (LPA) is a growth factor-like phospholipid that regulates various processes in the cell including cell motility, cell survival, cell proliferation, and cellular differentiation. LPA is produced from phospholipids by various enzymatic pathways. It works on specific G-protein coupled receptors called LPA receptors (LPARs). The signaling of LPARs leads to many actions on physiological, developmental, and pathological processes like liver fibrosis and liver cancer. It has been elucidated that fibrosis is firmly affected by elevated concentration of LPA and some of its receptors as LPAR1 and LPAR3 (Simo et al. 2014; Kaffe et al. 2017; Eraky et al. 2018; Xiang et al. 2020). Moreover, LPA signaling is involved in the fibrotic process through activating transforming growth factor-β1 (TGF-β1) which is considered as the cornerstone of fibrosis development (Li et al. 2017; Eraky et al. 2018).

The hepatic stellate cell (HSC) activation presents a substantial role in the initiation and development of liver fibrosis. Fibrogenic cytokines like TGF-β1 activate quiescent HSCs and promote trans-differentiation into myofibroblasts during liver injury. This step is characterized by the expression of α-smooth muscle actin (α-SMA), eventually leading to ECM accumulation (Higashi et al. 2017). TGF-β1 stimulates phosphorylation of small mothers against decapentaplegic (Smad)-2 and -3 which in turn triggers overexpression of pro-fibrotic genes such as collagen, α-SMA, and connective tissue growth factor (CTGF). TGF-β1/Smad pathway dysregulation is an important mechanism of pathogenesis in fibrosis of liver (Hu et al. 2018; Mu et al. 2018).

Ductular reaction is the proliferation of reactive bile ducts stimulated via liver injury. Cytokeratin 19 (CK19) is expressed normally in the lining of hepatobiliary tracts and considered as a helpful marker for the occurrence of ductular reaction which is closely related to liver fibrosis and damage (Jain et al. 2010; Sato et al. 2019).

Sesamol (SML), an active phenolic lignan and a prominent fragrance component in sesame oil, exhibits anti-oxidant, free radical scavenging ability (Gao et al. 2017) and anti-inflammatory effect (Abd Elrazik et al. 2021). Moreover, it has been demonstrated to display wide array of biological activities such as cardioprotective (Jayaraj et al. 2020), anti-mutagenic (Kaur and Saini 2000), radioprotective (Majdaeen et al. 2020), neuroprotective (Sachdeva et al. 2015), and anti-platelet activity (Chang et al. 2010). SML was reported to ameliorate cyclophosphamide-induced hepatotoxicity (Jnaneshwari et al. 2014), but the molecular mechanisms of anti-fibrotic effect of SML are not well known.

Therefore, the current study aimed to investigate the probable protective effects of SML against liver fibrosis induced in rats by TAA. Additionally, we also aimed to evaluate SML possible mechanisms of action.

## Materials and methods

### Chemicals

TAA and SML were purchased from Sigma-Aldrich (St. Louis, MO, USA). Other chemicals and reagents consumed during this study were of high analytical grade and purchased from El-Gomhouria Co. For Trading Drugs, Chemicals & Medical Supplies (Cairo, Egypt).

### Animals

Adult male Sprague–Dawley rats weighing 180–200 g (8–10 weeks old) were obtained from VACSERA CO, Egypt. The animals were allowed to standard rat pellet food and water ad libitum and housed under controlled condition of 25 ± 2 °C room temperature, 45–55% humidity, and 12-h dark/light cycle. This study protocol was approved by “Research Ethics Committee” Faculty of Pharmacy, Mansoura University, Mansoura City, Egypt (approval code: 2021–401), which is in accordance with “Guide for the Care and Use of Laboratory Animals, 8th edition” (National Research Council (US); Committee for the Update of the Guide for the Care and Use of Laboratory Animals, 2011).

### Experimental design

Rats were equally divided into four groups (8 animals/group):

**Control group**: rats received no treatment, only 0.2-mL sterile saline by intraperitoneal (i.p.) injection twice weekly for 8 weeks. **TAA group:** rats were injected by TAA (200 mg/kg dissolved in saline, i.p., twice weekly for 8 weeks) (Eraky et al. 2018). **SML 50 group:** rats received TAA (200 mg/kg/i.p.) twice weekly concurrently with SML (50 mg/kg/day) by oral gavage suspended in carboxymethyl cellulose (CMC) for 8 weeks (Hemalatha et al. 2013). **SML 100 group:** rats received TAA (200 mg/kg/i.p.) twice weekly concurrently with SML (100 mg/kg/day) by oral gavage suspended in CMC for 8 weeks (Hemalatha et al. 2013).

Based on previous studies and our preliminary experiments, it has been demonstrated that CMC had no statistically significant effects or treatment-related adverse effects and considered safe for all animal species (Helmy et al. 2019; Zeyada et al. 2020).

After 8 weeks, rats were weighted, anesthetized by thiopental (40 mg/kg, i.p.), and blood was gathered through the retro-orbital puncture and then centrifuged at 4000 rpm for serum separation and assessment of biochemical parameters. Then, all animals were sacrificed and their livers were isolated and weighted for calculation of the liver index (liver weight to body weight %) (Yogalakshmi et al. 2010). Isolated livers were divided into three portions. The first one was immediately transferred in RNA later, then stored at 4 °C overnight, and kept at − 80 °C for qRT-PCR. The second portion was homogenized in (10% w/v) phosphate buffered saline (pH 7.4) for oxidative stress marker assessment and ELISA experiment. The third portion was fixed in 10% neutral buffered formalin for histopathological and immunohistochemical analyses.

### Serum biochemical analysis

Serum was used to measure aspartate aminotransferase (AST) and alanine aminotransferase (ALT) activities (Spectrum Diagnostics, Egypt), in addition to measuring levels of albumin, total protein (BioMed Diagnostic, Egypt), and bilirubin (Diamond Diagnostics, Egypt) following the manufacturer’s instructions.

### Determination of oxidative stress biomarkers

Hepatic tissue supernatants were used to assess the tissue content of malondialdehyde (MDA) and glutathione (GSH) following the manufacturer’s instructions (Biodiagnostic, Giza, Egypt).

### Histopathological analysis of hepatic tissue

Paraffin-embedded blocks of hepatic tissue were dissected into 5-μm thickness segments, then stained with hematoxylin and eosin (H&E) stain for necroinflammatory scores assessment according to Ishak’s activity index (Ishak et al. 1995).

For analysis of collagen content in the fibrotic areas, sections were stained with Masson’s trichrome stain and the fibrosis intensity was demonstrated as a percentage of the fibrotic (stained) area to the total area via analyzing 30 random fields in the same slide (James et al. 1990).

### Immunohistochemistry

Immunohistochemical analysis was carried out on 5-μm-thick deparaffinized liver sections incubated overnight at 4 °C with polyclonal antibodies for TGF-β1 (Fine Test, China), CK19 (Zeta Corporation, USA), α-SMA, and vascular endothelial growth factor (VEGF) (Servicebio, China). Sections were treated with secondary antibodies conjugated with horseradish peroxidase and visualized by 2% diaminobenzidine reagent. Then, slides were counterstained with hematoxylin and examined by a light microscope.

### Determination of hepatic phosphorylated Smad3 (pSmad3) protein level by ELISA

Hepatic pSmad3 protein level was measured in the liver homogenates by commercially available ELISA kit (Bioassay, England) following manufacturer’s instruction.

### Gene expression analysis using quantitative real-time polymerase chain reaction (qRT-PCR)

RNA was isolated from liver tissues using Qiagen RNeasy Plus Mini kit (Qiagen®, USA). The concentration and purity of the extracted RNA were measured spectrophotometrically by NanoDrop System (Thermo Fisher Scientific Inc.). After that, RevertAid First Strand cDNA Synthesis Kit (Thermo Fisher Scientific, USA) was used to reverse transcript 2 μg of RNA to cDNA. Real-time PCR was carried out using *HERA* SYBR® Green qRT-PCR Kit (Willowfort, England) on Piko Real-PCR System (Thermo Fisher Scientific Inc.). The primer sequences used in this work are listed in Table 1. The mRNA expressions of these genes were normalized against glyceraldehyde 3-phosphate dehydrogenase (GAPDH) as a housekeeping gene and quantified via the 2^−ΔΔCt^ method.

**Table 1:** Sequences of primers used in qRT-PCR experiments

Name	Primer sequence
GAPDH — forward	5′-TCCCATTCTTCCACCTTTGA-3′
GAPDH — reverse	5′-CCACCACCCTGTTGCTGTAG-3′
LPAR1 — forward	5′-TTTCACAGCCCCAGTTCACA-3′
LPAR1 — reverse	5′-GCTTGCTCACAGTGTTCCAT-3′
LPAR3 — forward	5′-GCAAGGGTGGAGGTGTAGAA-3′
LPAR3 — reverse	5′-GGTCTAAACTCGCCATCACG-3′
CTGF — forward	5′-CGAGTCCTTCCAAAGCAGTT-3′
CTGF — reverse	5′-ATCACACACCCACTCCTCAC-3′
TGF-β1 — forward	5′-CCGCAACAACGCAATCTATGA-3′
TGF-β1 — reverse	5′-GCACTGCTTCCCGAATGTCT-3′
Smad3 — forward	5′-AGACACCAGTGCTACCTCCA-3′
Smad3 — reverse	5′-CCAGCGGGGAAGTTAGTGTT-3′
Smad7 — forward	5′-TTTTTCCCCCCACCCTTCCAAC-3′
Smad7 — reverse	5′-AAACACACCACCTTCTCGCACC-3′
α-SMA — forward	5′-ACCATCGGGAATGAACGCTT-3′
α-SMA — reverse	5′-CTGTCAGCAATGCCTGGGTA-3′
CK19 — forward	5′-CGCATCGTGTCCTCATCCTC-3′
CK19 — reverse	5′-GCCCCACTAAAACTTCCACC-3′

*GAPDH* glyceraldehyde 3-phosphate dehydrogenase, *LPAR1* lysophosphatidic acid receptor1, *LPAR3* lysophosphatidic acid receptor3, *CTGF* connective tissue growth factor, *TGF-β1* transforming growth factor β1, *Smad3* small mothers against decapentaplegic3, *Smad7* small mothers against decapentaplegic7, *α-SMA* alpha smooth muscle actin, *CK19* cytokeratin 19

### Statistical analysis

The experimental data were expressed as mean ± SEM. Data analysis and graphing were done by GraphPad Prism 6.01 (GraphPad Software, San Diego, CA, USA). One-way analysis of variance (ANOVA) followed by Tukey’s post hoc test was used for statistical evaluations. For the analysis of histopathology scores, non-parametric Kruskal–Wallis test followed by Dunn’s multiple comparison post hoc test were used.

## Results

### SML reduces liver index

Liver index significantly increased in TAA-intoxicated rats by 1.9-fold (*P* < 0.0001) compared with control group, while oral administration of either 50 or 100 mg/kg SML significantly reduced liver index by 23.7% (*P* < 0.0001) and 38.5% (*P* < 0.0001), respectively, compared with TAA group (Table 2).

**Table 2:** Effect of 50 and 100 mg/kg sesamol (SML) on liver index and liver function tests

Groups	Control	TAA	SML 50	SML 100
Liver index	2.1 ± 0.03	3.88 ± 0.04^++++^	3.02 ± 0.06^++++, ****^	2.43 ± 0.03^+++, ****, $$$$^
ALT (IU/L)	42.25 ± 4.18	192.62 ± 2.41^++++^	123.5 ± 3.7^++++, ****^	91.5 ± 1.45^++++, ****, $$$$^
AST (IU/L)	122.87 ± 3.36	416.5 ± 8.24^++++^	267.62 ± 3.34^++++, ****^	219.12 ± 3.45^++++, ****, $$$$^
Total bilirubin (mg/dL)	0.14 ± 0.01	0.66 ± 0.01^++++^	0.37 ± 0.02^++++, ****^	0.27 ± 0.01^++++, ****, $$$$^
Albumin (g/dL)	3.98 ± 0.03	2.75 ± 0.06^++++^	3.08 ± 0.03^++++, ****^	3.48 ± 0.04^++++, ****, $$$$^
Total protein (g/dL)	8.56 ± 0.11	6.21 ± 0.07^++++^	6.66 ± 0.05^++++, **^	7.05 ± 0.04^++++, ****, $$^

*ALT* alanine aminotransferase, *AST* aspartate aminotransferase, *TAA* thioacetamide

Values are expressed as mean ± SEM (*n* = 8). ^+^Significance against control group (^+++^*P* < 0.001, ^++++^*P* < 0.0001), ^*^significance against TAA group (^**^P < 0.01, ^****^P < 0.0001), and ^$^significance against SML 50 group (^$$^*P* < 0.01, ^$$$$^*P* < 0.0001)

### SML attenuates TAA-induced liver dysfunction and histopathological alterations in rats

TAA group showed a marked elevation in serum ALT and AST activities and total bilirubin level compared with control group (*P* < 0.0001). In contrary, albumin and total protein levels were significantly reduced in TAA group compared with control group (*P* < 0.0001). However, treating rats with either 50 or 100 mg/kg SML in the presence of TAA caused a significant improvement of liver function compared with TAA group (Table 2).

**Fig. 1:**
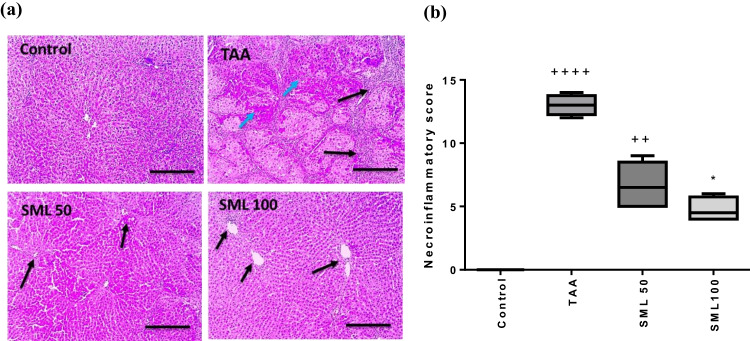
Sesamol (SML) attenuates TAA-induced histopathological alterations in rats. **a** Histopathological changes of liver sections stained with H&E. Blue arrows indicate necroinflammatory lesions and black arrows indicate fibrosis (× : 100, bar 100). **b** The necroinflammatory score. Values are expressed as median (*n* = 8). *TAA* thioacetamide. ^+^Significance against control group (^++^*P* < 0.01, ^++++^*P* < 0.0001). ^*^Significance against TAA group (.^*^P < 0.05)

Histopathological examination of liver tissues stained with H&E showed a normal architecture of liver tissues in control group, while TAA group showed a significant inflammation and necrosis compared with control group (*P* < 0.0001). On the contrary, both doses of SML treatment caused a reduction in necroinflammatory scores (Fig. 1).

### SML attenuates TAA-induced liver fibrosis

**Fig. 2:**
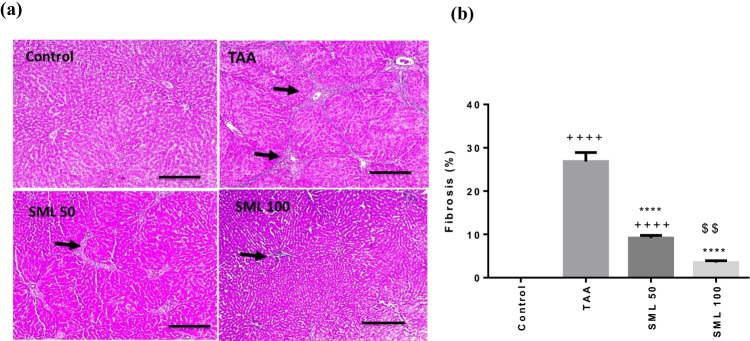
Sesamol (SML) attenuates TAA-induced liver fibrosis. **a** Liver sections stained with Masson’s trichrome. Black arrows indicate collagen deposition (× : 100, bar 100). **b** % fibrosis. *TAA* thioacetamide. Values are expressed as mean ± SEM (*n* = 6). ^+^Significance against control group (^++++^*P* < 0.0001), ^*^significance against TAA group (^****^P < 0.0001), and ^$^significance against SML 50 group (.^$$^*P* < 0.01)

In cross-sectional images of liver tissues stained with Masson’s trichrome, control group showed no collagen deposition, while TAA caused a remarkable increase in collagen deposition and fibrosis percentage in liver tissue (*P* < 0.0001). Rats treated with SML 50 or 100 mg/kg revealed a marked reduction in collagen deposition and fibrosis percentage compared with TAA-intoxicated rats (*P* < 0.0001) (Fig. 2).

### SML improves the anti-oxidant capacity in rats

**Fig. 3:**
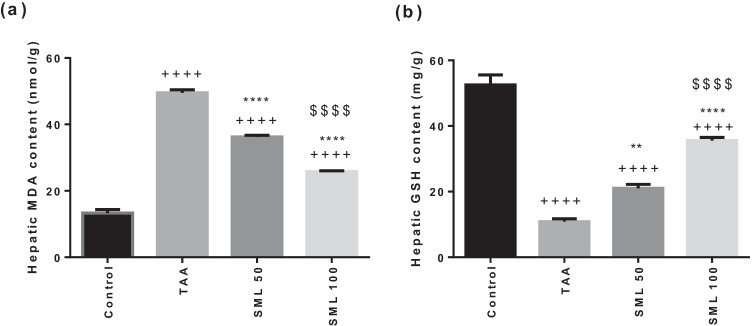
Sesamol (SML) improves the anti-oxidant capacity in rats. **a** Malondialdehyde (MDA). **b** Glutathione (GSH). *TAA* thioacetamide. Values are expressed as mean ± SEM (*n* = 8). ^+^Significance against control group (^++++^*P* < 0.0001), ^*^significance against TAA group (^**^P < 0.01, ^****^P < 0.0001), and ^$^significance against SML 50 group (.^$$$$^*P* < 0.0001)

As shown in Fig. 3, TAA group showed 3.7-fold (*P* < 0.0001) significant increase in hepatic MDA content associated with 4.7-fold (*P* < 0.0001) marked decrease in hepatic GSH content compared with control group. Interestingly, 50- or 100-mg/kg SML administration exhibited a dose-dependent decrease in MDA (*P* < 0.0001) as well as a dose-dependent elevation in GSH content (*P* < 0.01, *P* < 0.0001, respectively) compared with TAA group.

### SML down-regulates gene expressions of LPAR1, LPAR3, and CTGF

**Fig. 4:**
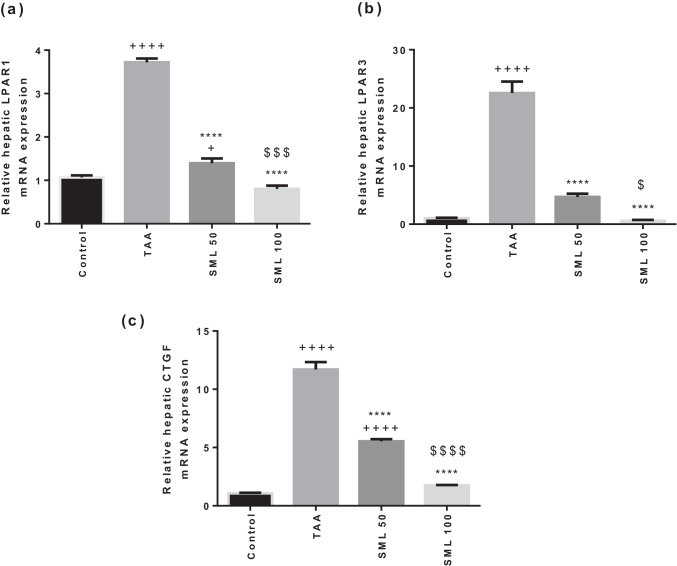
Sesamol (SML) down-regulates gene expressions of lysophosphatidic acid receptor1 (LPAR1), LPAR3, and connective tissue growth factor (CTGF). The mRNA expression of (**a**) LPAR1. (**b**) LPAR3. and (**c**) CTGF. *TAA* thioacetamide. Values are expressed as mean ± SEM (*n* = 6). ^+^Significance against control group (^+^*P* < 0.05, ^++++^*P* < 0.0001), ^*^significance against TAA group (^****^P < 0.0001), and ^$^significance against SML 50 group (^$^*P* < 0.05, ^$$$^*P* < 0.001, ^$$$$^*P* < 0.0001)

As shown in Fig. 4, TAA group showed a marked up-regulation in gene expressions of LPAR1, LPAR3, and CTGF compared with control group (*P* < 0.0001). However, treatment with SML 50 or 100 mg/kg caused a dose-dependent significant down-regulation in the mRNA expressions of LPAR1, LPAR3, and CTGF compared with TAA group (*P* < 0.0001).

### SML reduces TGF-β1 gene expression and its protein concentration

**Fig. 5:**
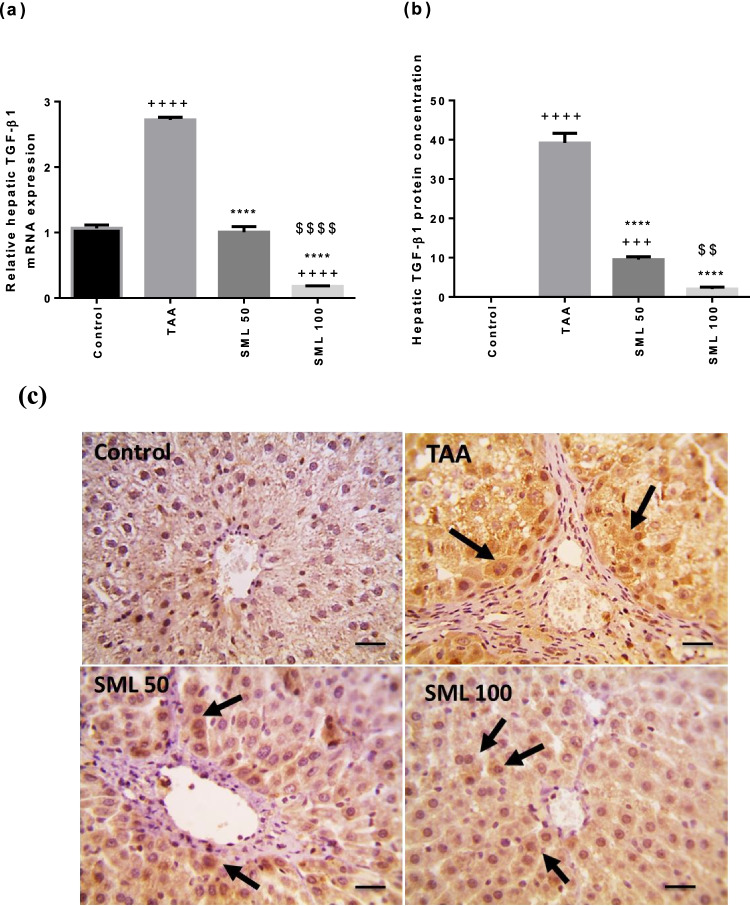
Sesamol (SML) reduces transforming growth factor β1 (TGF-β1) gene expression and its protein concentration. **a** The mRNA expression of TGF-β1. **b** and **c** The protein concentration of TGF-β1 using immunohistochemistry. Bars represent the number of positive cells per 10 high-power field in sections stained with anti-TGF-β1 antibodies; arrows indicate cytoplasmic protein expression of TGF-β1 that appears in hepatocytes around areas of fibrosis (× : 400, bar 50). *TAA* thioacetamide. Values are expressed as mean ± SEM (*n* = 6). ^+^Significance against control group (^+++^*P* < 0.001, ^++++^*P* < 0.0001), ^*^significance against TAA group (^****^P < 0.0001), and ^$^significance against SML 50 group (^$$^*P* < 0.01, ^$$$$^*P* < 0.0001)

As shown in Fig. 5, remarkable increase in TGF-β1 mRNA expression and its protein concentration was detected in TAA group, compared with control group (*P* < 0.0001). After 8 weeks of daily administration of either 50 or 100 mg/kg SML, gene expression and protein concentration of TGF-β1 (*P* < 0.0001) were reduced significantly compared with TAA group in a dose-dependent manner.

### SML down-regulates Smad3 gene expression and pSmad3 protein concentration and up-regulates Smad7 gene expression

**Fig. 6:**
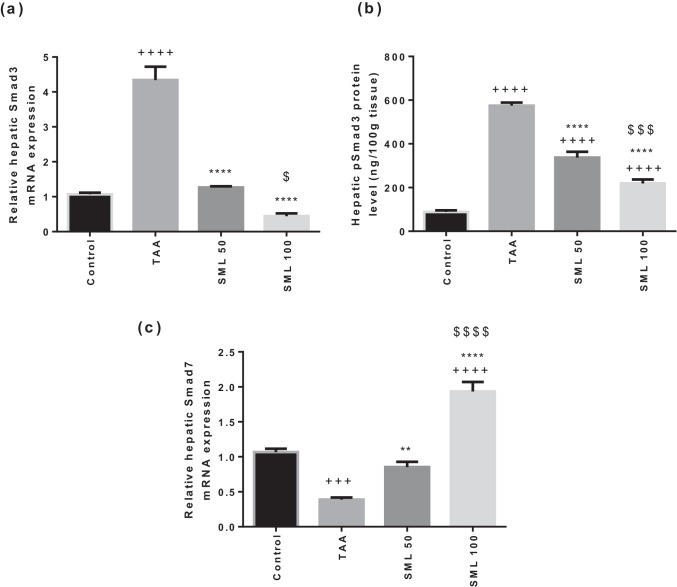
Sesamol (SML) down-regulates small mothers against decapentaplegic3 (Smad3) gene expression and phosphorylated Smad3 (pSmad3) protein concentration and up-regulates Smad7 gene expression. **a** The mRNA expression of Smad3 (*n* = 6). **b** The protein level of pSmad3 (*n* = 8). **c** The mRNA expression of Smad7 (*n* = 6). *TAA* thioacetamide. Values are expressed as mean ± SEM. ^+^Significance against control group (^+++^*P* < 0.001, ^++++^*P* < 0.0001), ^*^significance against TAA group (^**^P < 0.01, ^****^P < 0.0001), and ^$^significance against SML 50 group (^$^*P* < 0.05, ^$$$^*P* < 0.001, ^$$$$^*P* < 0.0001)

TAA group showed about 4.3-fold (*P* < 0.0001) significant elevation in Smad3 gene expression as well as 6.5-fold (*P* < 0.0001) marked increase in pSmad3 protein level compared with control group. However, SML 50- and 100-mg/kg treatments showed a dose-dependent marked decrease in the mRNA expression of Smad3 and pSmad3 protein level compared with TAA group (*P* < 0.0001) (Fig. 6a, b).

Gene expression of Smad7 significantly down-regulated by 70% (*P* < 0.001) in TAA group compared with control group. On the contrary, treatment with SML either 50 or 100 mg/kg revealed 2.6- (*P* < 0.01) and 6.3-fold (*P* < 0.0001) marked increase in Smad7 mRNA expression, respectively, compared with TAA group in a dose-dependent manner (Fig. 6c).

### SML suppresses α-SMA gene expression and its protein concentration

**Fig. 7:**
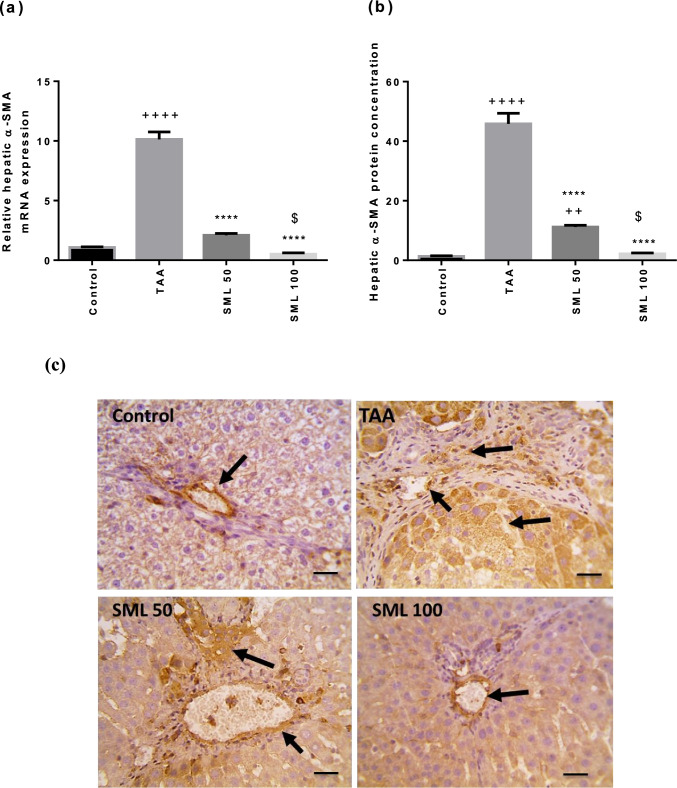
Sesamol (SML) suppresses alpha smooth muscle actin (α-SMA) gene expression and its protein concentration. **a** The mRNA expression of α-SMA. **b** and **c** The protein concentration of α-SMA using immunohistochemistry. Bars represent the number of positive cells per 10 high-power field in sections stained with anti-α-SMA antibodies; arrows indicate positively stained cells (× : 400, bar 50). *TAA* thioacetamide. Values are expressed as mean ± SEM (*n* = 6). ^+^Significance against control group (^++^*P* < 0.01, ^++++^*P* < 0.0001), ^*^significance against TAA group (^****^P < 0.0001), and ^$^significance against SML 50 group (.^$^*P* < 0.05)

TAA significantly increased α-SMA gene expression and its protein concentration compared with control group (*P* < 0.0001). Administration of SML either 50 or 100 mg/kg caused a dose-dependent marked decrease in the mRNA expression and protein concentration of α-SMA compared with TAA group (*P* < 0.0001) (Fig. 7).

### SML decreases CK19 gene expression and its protein concentration

**Fig. 8:**
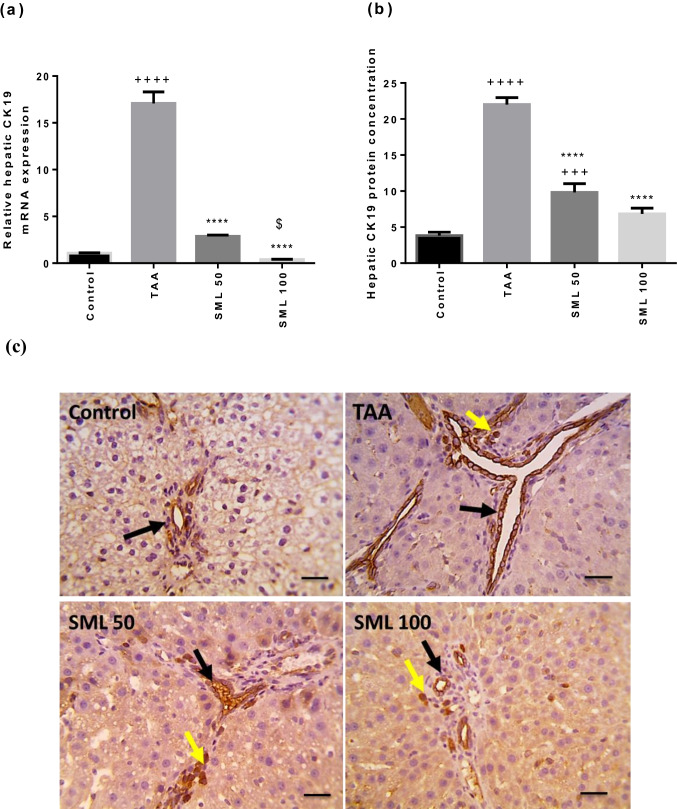
Sesamol (SML) decreases cytokeratin 19 (CK19) gene expression and its protein concentration. **a** The mRNA expression of CK19. **b** and **c** The protein concentration of CK19 using immunohistochemistry. Bars represent the number of positive cells per 10 high-power field in sections stained with anti-CK19 antibodies; black arrows indicate positively stained bile ducts and yellow arrows indicate macrophage that infiltrates the fibrous septa (× : 400, bar 50). *TAA* thioacetamide. Values are expressed as mean ± SEM (*n* = 6). ^+^Significance against control group (^+++^*P* < 0.001, ^++++^*P* < 0.0001), ^*^significance against TAA group (^****^P < 0.0001), and ^$^significance against SML 50 group (.^$^*P* < 0.05)

TAA caused a remarkable up-regulation in gene expression of CK19 compared with control group (*P* < 0.0001). Treatment with 50 or 100 mg/kg SML showed a dose-dependent significant down-regulation in CK19-mRNA expression compared with TAA group (*P* < 0.0001) (Fig. 8a). Immunohistochemical analysis revealed positively stained bile ducts in control group and a significant increase and expansion of CK19-positive staining in portal areas in TAA group compared with control group (*P* < 0.0001) due to proliferation of bile ductules and macrophage infiltrates the fibrous septa. Interestingly, both SML groups showed a marked decrease in CK19 protein concentration compared with TAA group (*P* < 0.0001) (Fig. 8b, c).

### SML reduces VEGF protein concentration

**Fig. 9:**
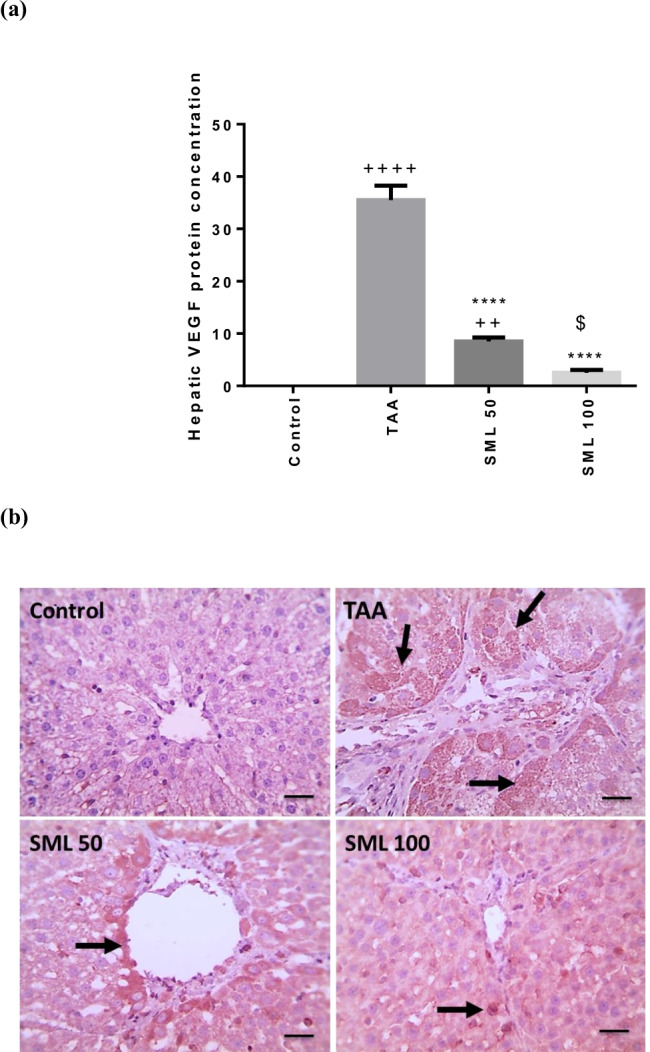
Sesamol (SML) reduces vascular endothelial growth factor (VEGF) protein concentration. **a** and **b** The protein concentration of VEGF using immunohistochemistry. Bars represent the number of positive cells per 10 high-power field in sections stained with anti-VEGF antibodies; arrows indicate positively stained cells (× : 400, bar 50). *TAA* thioacetamide. Values are expressed as mean ± SEM (*n* = 6). ^+^Significance against control group (^++^*P* < 0.01, ^++++^*P* < 0.0001), ^*^significance against TAA group (^****^P < 0.0001), and ^$^significance against SML 50 group (.^$^*P* < 0.05)

Protein concentration of VEGF markedly increased in TAA group compared with control group (*P* < 0.0001), while treatment with SML 50 or 100 mg/kg exhibited a significant reduction in VEGF protein concentration (*P* < 0.0001) compared with TAA group in a dose-dependent manner (Fig. 9).

## Discussion

Liver fibrosis is a threatening health problem leading to development of cirrhosis and liver cancer (Zhang et al. 2020). Induction of liver fibrosis by TAA is considered as a well-established model for evaluation of potential anti-fibrotic drugs as it produces hepatic disease in rodents resemble to those observed in humans (Yanguas et al. 2016).

In our study, TAA group showed a marked increase in liver index due to ECM accumulation in liver leading to an increase in relative liver weight. Serum ALT and AST activities were elevated in TAA group due to centrilobular necrosis caused by TAA that was accompanied by hepatic enzyme leakage in the serum. Additionally, serum total bilirubin was increased upon TAA injection due to impairment of liver detoxifying function. Moreover, TAA caused a remarkable decrease in serum albumin and total protein due to attenuation of synthetic function of the hepatocytes; these results are in accordance with many studies (Czechowska et al. 2015; El-Mihi et al. 2017). SML administration caused improvement and restoration of hepatic functions, particularly in rats treated with the higher dose of SML (100 mg/kg). These results were further reinforced via the histopathological examination of liver tissues which indicated the ability of SML to protect against the pathological alterations in the liver architecture and collagen deposition after exposure to TAA.

Oxidative stress has been reported as a main molecular mechanism implicated in liver fibrosis induced by TAA. Releasing ROS and toxic metabolites after the metabolism of TAA in liver resulted in hepatocyte necrosis, lipid peroxidation of cell membrane leading to MDA release, and imbalance of anti-oxidant defense system due to depletion of GSH (Tsai et al. 2010; Sukalingam et al. 2018). SML, particularly high-dose, revealed anti-oxidant properties as it abolished MDA accumulation and GSH reduction. This is in accordance with the reported anti-oxidant activity of SML that might be attributed to the up-regulation in the anti-oxidant enzymes such as catalase and superoxide dismutase (Gao et al. 2017; Majdalawieh and Mansour 2019; Abd Elrazik et al. 2021).

Treatment of rats with TAA increases autotaxin expression which in turn hydrolyzes lysophosphatidyl choline (LPC) into LPA (Lebda et al. 2018; Kaffe et al. 2019). LPA is a pro-fibrogenic factor in liver that is derived through autotaxin from hepatocytes and acts in a paracrine way in order to induce activation and proliferation of adjacent HSCs via binding to LPARs (1 and 3) (Kaffe et al. 2017; Eraky et al. 2018). It has been reported that LPA-LPAR1/LPAR3 signaling is included in fibrosis of liver and other tissues like renal and lung fibrosis (Pradère et al. 2008; Shea and Tager 2012; Kaffe et al. 2019). Our results showed significant elevation in LPAR1/LPAR3 expression in TAA-intoxicated rats. So, LPAR1/LPAR3 down-regulation could provide therapeutic benefits against hepatic fibrosis. According to our results, SML treatment showed marked down-regulation of LPAR1 and LPAR3 gene expressions in a dose-dependent manner proposing that SML might exert its anti-proliferative and anti-fibrogenic effects via targeting LPAR1/LPAR3 signaling pathway. These results are in agreement with previous study which correlated the protective effect against hepatic fibrosis with the down-regulation of LPARs (Eraky et al. 2018). To our knowledge, this study is the first one to investigate the role of SML in LPAR1/LPAR3 expression as a possible mechanism of action of its protective role against TAA-induced liver fibrosis.

CTGF is an important downstream molecule in the LPA-LPAR1/LPAR3 and TGF-β1 signaling pathways. CTGF up-regulation is considered as a central pathway participating in activation of HSCs during hepatic fibrosis (Cabello-Verrugio et al. 2011; Gan et al. 2011; Weiskirchen 2011; Ramazani et al. 2018). Induction of liver fibrosis by TAA causes a marked elevation in CTGF expression and that was in agreement with previous studies (Park et al. 2016; Algandaby et al. 2017; Eraky et al. 2018). SML, especially the high dose, markedly attenuated the increase of CTGF expression in TAA rats that might explain its anti-fibrotic effect.

TGF-β1 is a chief moderator in the development of liver fibrosis and a promising target to treat fibrosis. It is produced by hepatocytes, sinusoidal endothelial cells, and Kupffer cells in response to liver injury (Kajdaniuk et al. 2013; El-Mowafy et al. 2021). Additionally, LPARs are involved in the induction of TGF-β expression (Li et al. 2017). TGF-β1 is an important factor in the activation of HSCs. Binding of active TGF-β1 to its receptors results in phosphorylation of cytoplasmic mediators Smad2 and Smad3 and consequently a Smad complex is formed with Smad4. Afterward, Smad complex translocates into the nucleus to control the transcription of the target genes such as α-SMA and CTGF (Liu and Desai 2015). Activated HSCs express large amounts of α-SMA which is a unique marker for HSCs. Smad7 negatively regulates TGF-β1/Smad3 signaling via binding to TGF-β type I receptors and blocking Smad2/3 phosphorylation (Hu et al. 2018). Fibrotic cascade is triggered by activation of TGF-β1/Smad3 pathway or loss of inhibitory Smad such as Smad7 (Hu et al. 2018; Dewidar et al. 2019). Our study showed stimulation of TGF-β1/Smad3 pathway in TAA group as indicated by an increase in TGF-β1, Smad3, pSmad3, α-SMA expressions, and down-regulation of Smad7 expression. On the other hand, the enhanced TGF-β1, Smad3, pSmad3, and α-SMA in TAA rats were dose-dependently attenuated after SML treatment in addition to elevation in the inhibitory Smad7. These results suggest that SML inhibits HSC activation by targeting TGF-β1/Smad3 pathway.

Although the activated HSC is the key effector cell type in liver fibrosis (Trautwein et al. 2015), there is another cell that can remarkably participate in fibrosis, including hepatic progenitor cells (HPCs) (Yovchev et al. 2008; Wang et al. 2009). Ductular reaction is the appearance of proliferating bile ductular structures upon liver injury (Sato et al. 2019). These proliferating ductules are assumed to emerge from HPCs (Falkowski et al. 2003; Roskams 2003; Roskams et al. 2004). A strong correlation between fibrosis extent and ductular reaction has been documented in previous studies (Clouston et al. 2005; Wood et al. 2014). The present study revealed that the gene expression and protein concentration of CK19, marker of HPCs and ductular reaction, was remarkably increased in the TAA group, but reduced after treatment with SML suggesting its ability to ameliorate ductular reaction.

Inflammation and hypoxia are important elements in angiogenesis. Liver tissue impacted by fibrosis exhibits a persistent inflammation and low level of oxygen due to ECM accumulation in liver parenchyma and disrupted blood vessels, leading to rise in major angiogenic factor (VEGF) expression and neovascularization (Elpek 2015; Zadorozhna et al. 2020). Bile ductules are continuously associated with microvessels, and ductule number matches with accompanied microvessel density, which gives an indication about the link between ductular reaction and microvessels in addition to angiogenesis (Gouw et al. 2006). Our study showed a marked elevation in VEGF protein concentration in TAA rats that was in accordance with other studies (Nakamura et al. 2014; Algandaby et al. 2017; Elnfarawy et al. 2021). However, we found that treating TAA rats with SML significantly decreased the protein concentration of VEGF that may indicate its possible anti-angiogenic activity.

## Conclusion

Sesamol alleviated hepatic fibrosis induced by TAA in rats. The hepatoprotective effect of SML may be attributed to down-regulation of LPAR1/3 expression, inhibition of TGF-β1/Smad3 signaling, up-regulation of Smad7 expression, and eventually decreasing ductular reaction and angiogenesis.

## Supplementary Information

Below is the link to the electronic supplementary material.

Supplementary file1 (XLSX 25 KB)

Supplementary file2 (XLSX 16 KB)

## Data Availability

All data generated or analyzed during this study are included in this article (and its supplementary information files).

## Abbreviations

α-SMA: α-Smooth muscle actin
CTGF: Connective tissue growth factor
CK19: Cytokeratin 19
CMC: Carboxy methyl cellulose
GAPDH: Glyceraldehyde 3-phosphate dehydrogenase
ECM: Extracellular matrix
GSH: Glutathione
H&E: Hematoxylin and eosin
HPCs: Hepatic progenitor cells
HSCs: Hepatic stellate cells
LPAR: Lysophosphatidic acid receptor
MDA: Malondialdehyde
ROS: Reactive oxygen species
Smad: Small mothers against decapentaplegic
SML: Sesamol
TAA: Thioacetamide
TGF-β1: Transforming growth factor-β1
VEGF: Vascular endothelial growth factor
